# Eating locally: Australasian gannets increase their foraging effort in a restricted range

**DOI:** 10.1242/bio.013250

**Published:** 2015-09-14

**Authors:** Lauren P. Angel, Sophie Barker, Maud Berlincourt, Emma Tew, Victoria Warwick-Evans, John P. Y. Arnould

**Affiliations:** 1 School of Life and Environmental Sciences, Deakin University, 221 Burwood Hwy, Burwood, Victoria 3125, Australia; 2 School of Environmental Sciences, University of Liverpool, L69 3GP, UK

**Keywords:** Foraging ecology, Inter-annual, Accelerometry, *Morus serrator*

## Abstract

During the breeding season, seabirds adopt a central place foraging strategy and are restricted in their foraging range by the fasting ability of their partner/chick and the cost of commuting between the prey resources and the nest. Because of the spatial and temporal variability of marine ecosystems, individuals must adapt their behaviour to increase foraging success within these constraints. The at-sea movements, foraging behaviour and effort of the Australasian gannet (*Morus serrator*) was determined over three sequential breeding seasons of apparent differing prey abundance to investigate how the species adapts to inter-annual fluctuations in food availability. GPS and tri-axial accelerometer data loggers were used to compare the degree of annual variation within two stages of breeding (incubation and chick rearing) at a small gannet colony situated between two larger, nearby colonies. Interestingly, neither males nor females increased the total distance travelled or duration of foraging trip in any breeding stage (*P*>0.05 in all cases) despite apparent low prey availability. However, consistently within each breeding stage, mean vectorial dynamic body acceleration (an index of energy expenditure) was greater in years of poorer breeding success (increased by a factor of three to eight), suggesting birds were working harder within their range. Additionally, both males and females increased the proportion of a foraging trip spent foraging in a poorer year across both breeding stages. Individuals from this colony may be limited in their ability to extend their range in years of low prey availability due to competition from conspecifics in nearby colonies and, consequently, increase foraging effort within this restricted foraging area.

## INTRODUCTION

Individuals provisioning young at a natal site are limited in their foraging range by the cost of transport and/or the fasting ability of, and predation risk to, the unattended offspring (Orians and Pearson, 1979). Consequently, profitable prey patches which occur close to the central location will be targeted first as they provide the highest rate of net energy intake versus effort expended and minimise the time away from the offspring (Pyke et al., 1977). However, in periods of reduced prey availability, central place foragers will have to increase effort and/or extend their range in search of resources to meet the nutritional needs of the offspring and their own maintenance (Cairns, 1987; Abrams, 1991).

Marine central place foragers, such as breeding seabirds, are subject to high spatial and temporal variability in their environment (Weimerskirch, 2007). Changes in sea-surface temperature, wind stress and ocean circulation can all alter prey distribution at multiple spatial and temporal scales (Montevecchi, 1993), and individuals must adapt their behaviour in order to maximise foraging success and, consequently, breeding performance (Inchausti et al., 2003; Sandvik et al., 2012; Watanuki and Ito, 2012). For example, as seen in common murres (*Uria aalge*) in Canada, individuals may increase their foraging range in response to environmental variability, flying further from the colony in years when preferred prey is low (Burke and Montevecchi, 2009). Alternatively, common murres in Scotland switched to more predictable but less energy-dense prey (Wanless et al., 2005) while black-browed albatross (*Thalassarche melanophris*) have been shown to increase the frequency of chick-provisioning to compensate for smaller, less available prey (Weimerskirch et al., 1997).

In addition, it has long been hypothesised that neighbouring colonies of conspecifics will spatially segregate in foraging areas to reduce intra-specific competition and that colony size may be limited by available habitat within the maximum foraging range (Adams, 2001). Indeed, Wakefield et al. (2013) recently documented how northern gannet (*Morus bassanus*) populations around the United Kingdom forage in largely mutually exclusive areas despite their potential home ranges overlapping. However, while density-dependent competition at each colony will influence the extent of an individuals' foraging range, geographic boundaries (e.g. continental shelf-edge, protruding coastlines), in conjunction with proximity to other colonies, may restrict the ability of individuals to extend their range in times of reduced prey availability. Such restrictions could lead to differential reproductive responses to environmental change between colonies and, ultimately, influence population trajectories.

The Australasian gannet (*Morus serrator*) is a large marine predator breeding in colonies of 10–12,300 nests in New Zealand and Australia (Nelson, 1978; Bunce et al., 2002). As with other Sulids (gannets and boobies), the species forages mainly on pelagic schooling fish, primarily by plunge diving, often feeding in association with conspecifics and heterospecific competitors (Bunce and Norman, 2000). In south-eastern Australia, Australasian gannets forage on the shallow (and in places, narrow) continental shelf region of Bass Strait, located between mainland Australia and Tasmania, playing an important role in the ecosystem (Bunce, 2001). Bass Strait is influenced by warm surface waters and cool, deep Antarctic and sub-Antarctic waters (Gibbs, 1992), as well as the seasonally strong Bonney Upwelling (Nieblas et al., 2009). Inter-annual variability in the environmental conditions of Bass Strait has been shown to influence the diet, foraging behaviour and breeding performance of marine predators in the region (Mickelson et al., 1992; Gibbens and Arnould, 2009; Ropert-Coudert et al., 2009a; Hoskins and Arnould, 2014; Knox et al., 2014).

While other Sulids have been shown to increase their foraging range (Hamer et al., 2007; Hennicke and Weimerskirch, 2014), alter their diet (Pichegru et al., 2007) and fly towards previously successful areas (Weimerskirch et al., 2005a) when local conditions are poor, little is known about the behavioural response of Australasian gannets to environmental variability. Whereas Bunce and Norman (2000) found that Australasian gannets altered their diet during a large scale mortality of their preferred prey, the impact on the colony was only short-term (Pyk et al., 2013). As south-eastern Australia is predicted to experience substantial oceanographic changes in coming decades (Poloczanska et al., 2007; Ridgway and Hill, 2009), information regarding behavioural adaptation to persisting environmental change is necessary to predict the response of the population. This is especially so in view of the precarious nature of some colonies (i.e. small colony size; Norman et al., 1998), the added impact of fisheries interactions and other detrimental anthropogenic effects (Bunce, 2001), and the important ecological role (and economic significance in ecotourism) some colonies may have in unique localised habitats (Edmunds, 2003). Therefore, the aims of the present study were to determine whether Australasian gannets alter their: (1) at-sea movements; and/or (2) foraging effort in response to variability in a proxy for local prey conditions.

## RESULTS

There were significant differences in the fledging success of gannets between the three years of the study (Table 1), decreasing from 64.7% in 2011 to 30.1% in 2012 and 8.62% in 2013 (Chi-squared test: χ^2^=46.45, *P*<0.001). These findings coincided with variations in environmental conditions in the region. Although sea surface temperature measured within Bass Strait and Port Phillip Bay, was not significantly different across the three years during the breeding months (One-way ANOVA: *F*_2,28_*=*0.71, *P*=0.5; *F*_2,28_=0.67, *P*=0.5; Table 1). During the winter months preceding breeding, both sea surface temperatures (SST) and chlorophyll-*a* (chl-*a*) concentrations in Bass Strait (*F*_2,39_=8.89, *P*<0.001; *F*_2,39_=7.08, *P*=0.002) and Port Phillip Bay (*F*_2,39_=4.33, *P*<0.02; *F*_2,38_=16.31, *P*<0.001) were significantly different, with colder temperatures and higher chl-*a* concentrations occurring in years when fledging success was highest (64.7% breeding success; Table 1). Furthermore, in the Bonney Upwelling during January-March prior to the breeding season, average SST significantly increased (from 17.4±0.2°C in 2011 to 19.1±0.2°C in 2013; *F*_2,36_=12.39, *P*<0.001) and chl-*a* significantly reduced (from 0.29±0.02 mg m^−3^ in 2011 to 0.19±0.01 mg m^−3^ in 2013; *F*_2,36_=12.51, *P*<0.001), corresponding with years when breeding success was lower (Table 1). Consequently, these observations suggest that environmental conditions across the three years may have resulted in a decrease in food availability during the breeding seasons investigated which negatively impacted the birds' ability to raise offspring.

**Table 1.**
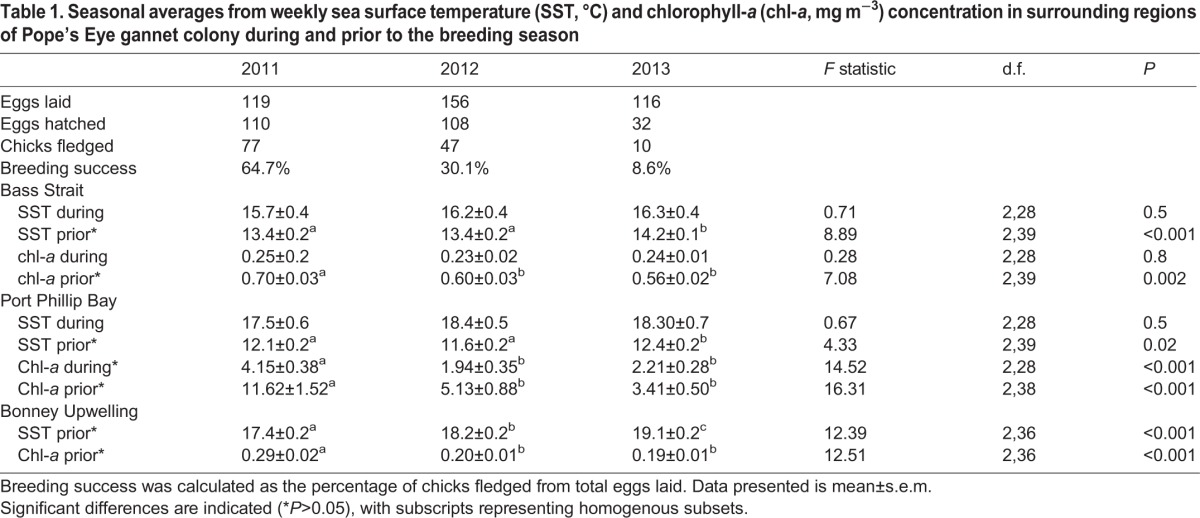
**Seasonal averages from weekly sea surface temperature (SST, °C) and chlorophyll-*a* (chl-*a*, mg m^−3^) concentration in surrounding regions of Pope's Eye gannet colony during and prior to the breeding season**

A total of 103 Australasian gannets were tracked from Pope's Eye across the three consecutive years (2011, *n*=32; 2012, *n*=46; 2013, *n*=25). Because so few nests successfully hatched chicks in 2013, no data were obtained during chick rearing of that year. Furthermore, due to device failure in incubation 2011, of the 15 devices deployed, GPS and acceleration data were only available for 2 males and 4 females. Hence, results incorporating incubation 2011 should be interpreted cautiously. The total distance travelled during a foraging trip had a significant difference between sex (*F*_1,99_=8.56, *P*<0.01) and stage (*F*_1,99_=4.58, *P*<0.05). Hence, inter-annual variability was analysed separately for each sex. While a high percentage of birds sampled were from pairs (44% in 2011, 74% in 2012, and 72% in 2013), no correlations were found between breeding pairs in foraging behaviour (Pearson correlation: foraging range: *r*^2^=0.03; distance travelled: *r*^2^=0.003; trip duration: *r*^2^=0.02, *P*>0.05 in all cases) or effort (total VeDBA: *r*^2^=0.05; mean VeDBA: *r*^2^=0.4, *P*>0.05 in all cases). Thus, for the purposes of this study individuals within pairs were considered independent from each other.

**Fig. 1.**
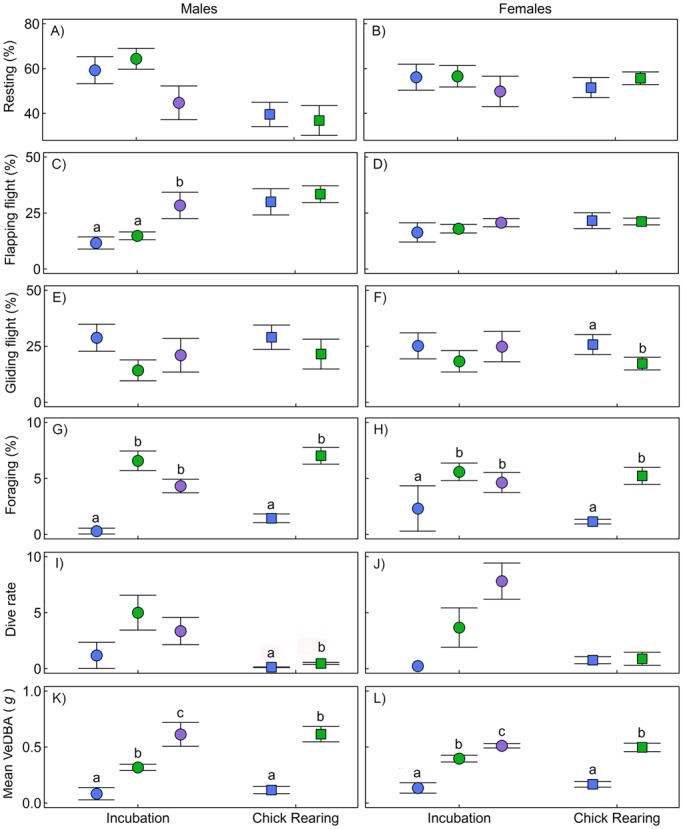
**Comparison of foraging behaviour and effort for Australasian gannets across three years.** Behaviours include proportion of foraging trip spent resting on the sea surface (A,B); flapping flight (C,D); gliding flight (E,F); foraging (G,H); and dive rate (dives h^−1^) (I,J). Energy expenditure is represented by mean vectorial dynamic body acceleration (VeDBA) (***g***) (K,L). In variables with significant results (*P*>0.05) homogenous subsets are indicated by superscripts. Data are represented as mean±s.e.m. 2011, blue; 2012, green; 2013, purple. Breeding stages: incubation, circles; chick rearing, squares.

There were no significant differences between years in the total distance travelled, maximum distance from the colony or duration of the foraging trip in both breeding stages (*P*>0.05 in all cases; Table 2) for either sex. While males travelled at a greater average speed during incubation in 2011 (14.2±3.5 km h^−1^) compared to 2012 (6.9±1.0 km h^−1^) and 2013 (7.0±0.6 km h^−1^; *F*_1,21_=3.5, *P*<0.05), average speed did not increase in any other stage of breeding in years of lower apparent food availability. During this stage, males spent a significantly greater proportion of the foraging trip flapping in 2013 (28.4±5.9%) compared to 2011 (11.6±2.7%) and 2013 (14.8±1.7%; *F*_2,16_=5.05, *P*<0.05; Fig. 1C). However, males did not significantly differ in the proportion of foraging trip spent gliding (*F*_2,16_=0.01, *F*_1,16_=1.69; Fig. 1E) or resting (*F*_2,16_=3.35, *F*_1,16_=0.10; Fig. 1A) between years, in either stage (*P*>0.05 all cases). Females spent a greater proportion of their foraging trip gliding in 2011 (25.8±2.0%) compared to 2012 (17.3±1.9%; *F*_1,23_=11.98, *P*<0.01; Fig. 1F), during chick rearing. However, they did not significantly increase the proportion of the foraging trip spent flapping (*F*_2,16_=0.47, *F*_1,23_=0.02, Fig. 1D) or resting (*F*_2,16_=0.32, *F*_1,23_=0.65, Fig. 1A) across years, in incubation or chick rearing, respectively (*P*>0.05 all cases).

**Table 2.**
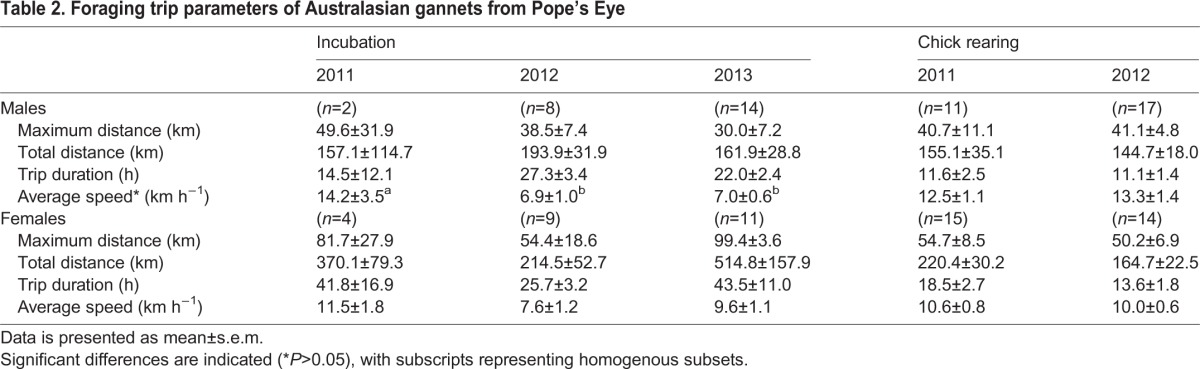
**Foraging trip parameters of Australasian gannets from Pope's Eye**

In contrast, the proportion of time at sea spent foraging increased in 2012, a year of lower prey availability, for males during incubation (0.3±0.3% in 2011 compared to 6.6±0.9% in 2012; *F*_2,16_=14.58, *P*<0.05) and chick rearing (1.4±0.4% in 2011 compared to 8.3±1.4% in 2012; *F*_1,16_=25.73, *P*<0.05). However, the time spent foraging did not significantly differ between 2012 and 2013 for incubation (*P*=0.74, Fig. 1G). Females also increased the proportion of time spent foraging from 2011 to 2012, in incubation (2.3±2.0% to 7.1±1.2%; *F*_2,16_=7.83, *P*<0.01) and chick rearing (1.1±0.2% to 5.8±0.9%; *F*_1,23_=13.45, *P*<0.01), but not in incubation between 2012 and 2013 (*P*=0.66, Fig. 1H). Dive rate was only significantly greater for males in 2012 during chick rearing (0.1±0.03 dives h^−1^ in 2011 to 0.5±0.1 in 2012 to; *F*_1,16_=12.90; Fig. 1I). In no other stages did males or females increase their dive rate.

Mean VeDBA (an index of energy expenditure) increased by a factor of three to eight (depending on stage) for both sexes, suggesting a higher rate of foraging effort, during years of presumably lower prey availability (*P*<0.001 in all cases; Fig. 1K,L). Total energy expended (total VeDBA) was significantly greater in poorer years. Total VeDBA during incubation increased from 1.7×10^5^±1.6×10^5^ ***g*** and 1.9×10^5^±0.7×10^5^ ***g*** in 2011, to 7.9×10^5^±0.9×10^5^ ***g*** and 6.6×10^5^±1.4×10^5^ ***g*** in 2012, to 10.0×10^5^±1.3×10^5^ ***g*** and 28.0×10^5^±6.1×10^5^ ***g*** in 2013 for males (*F*_2,16_=5.67, *P*<0.05) and females (*F*_2,16_=17.64, *P*<0.001), respectively. During chick rearing total VeDBA increased from 1.5×10^5^±0.6×10^5^ ***g*** and 3.3×10^5^±1.1×10^5^ ***g*** in 2011 to 3.3×10^5^±1.1×10^5^ ***g*** and 6.1×10^5^±1.0×10^5^ ***g*** in 2012 for males (*F*_1,16_=11.98, *P*<0.01) and females (*F*_1,23_=7.81, *P*<0.01), respectively.

## DISCUSSION

In years of reduced local food availability, numerous seabirds have been shown to extend their foraging range to acquire sufficient resources for chick provisioning and self-maintenance (Cairns, 1987). As central place foragers, however, breeding seabirds are restricted in their foraging range due to the fasting capability of either their partner, during incubation, or their offspring, during chick rearing (Ricklefs et al., 1985; Clutton-Brock, 1991). Should environmental conditions or prey availability drop below the critical threshold, long-lived birds typically prioritise their own survival resulting in a decrease in breeding success (Erikstad et al., 1998). In the present study, the breeding success in 2011 (64.7%) was similar to the historical average (63.4±7.2% between 1988 and 2006; Pyk et al., 2013) however it dropped substantially in the two subsequent years which coincided with poor environmental conditions consistent with reduced prey availability (Stenseth et al., 2002). Analysis of the foraging behaviour by Australasian gannets from this colony across the three breeding seasons suggests that, unlike observations in most seabirds, individuals did not increase their foraging range in response to apparent reduced prey availability but instead increased effort within their restricted range.

Consistent with that observed in other species (Thompson and Ollason, 2001; Monticelli et al., 2007), the results of the present study found indices of marine primary productivity in regions adjacent to the colony in the months preceding the breeding season were higher in years of greater reproductive success. As has been proposed for species elsewhere (Frederiksen et al., 2004; Cullen et al., 2009), such relationships could be used to broadly predict fledging success in Australasian gannets at this and other colonies. In the winter months prior to the breeding season sea surface temperature was lower and chlorophyll-*a* was higher in the regions where the gannets forage (Bass Strait and Port Phillip Bay). Furthermore, fluctuation in indices of primary productivity in the Bonney Upwelling (500 km to the west) 8–10 months prior to the breeding season were found to correspond to differences in fledging success. A similar time lag of the influence of the Bonney Upwelling has previously been observed in Australian fur seal breeding in central northern Bass Strait (Gibbens and Arnould, 2009). These results highlight the importance of this seasonally active upwelling in influencing the nutrient cascade, numerous trophic levels and top predators of the region. Indeed, in 2013 low breeding success was also observed in other Bass Strait marine predators such as short-tailed shearwaters *Puffinus tenuirostris* (Berlincourt and Arnould, 2015b), little penguins *Eudyptula minor* (Berlincourt and Arnould, 2015a), and Australian fur seals *Arctocephalus pusillus doriferus* (J.P.Y.A., unpublished data), indicative of poor foraging conditions.

Throughout periods of reduced food availability increased foraging range and duration has been observed in numerous seabirds (Hamer et al., 1993; Monaghan et al., 1994; Ronconi and Burger, 2008). In the Sulidae, Abbot's booby (*Papasula abbotti*) and northern gannets have been found to perform longer foraging trips in response to variable environmental conditions (Hamer et al., 2007; Hennicke and Weimerskirch, 2014). Pichegru et al. (2010), however, found that while Cape gannets (*M. capensis*) did not travel further from the colony when local resources decreased, individuals increased their foraging trip duration and total distance travelled in search of prey within their foraging range and also switched to less energy dense prey, provided by fisheries discards (Pichegru et al., 2007). A similar reliance on fisheries discards has been observed in northern gannets in times of poor food conditions (Votier et al., 2013), an option less available to Australasian gannets in Bass Strait due to the smaller scale of commercial fisheries operating there (Bunce, 2001). In the present study, in years of apparent reduced food availability neither male nor female Australasian gannets increased trip duration, maximum range or total distance travelled. Indeed, 97% of birds remained within 150 km of the colony, similar to previous observations for individuals at the study colony (Pyk, 2012), with only 6% of birds foraging for longer than 48 h in the poor years. While, Cape and northern gannets, from colonies of a similar size to Pope's Eye, have been found to forage 114–160 km from the colony (Grecian et al., 2012; Ludynia et al., 2012), it is expected that in years of presumably poor prey availability, gannets would forage to their maximum potential. Australasian gannets are capable of foraging up to 550 km from the colony (Machovsky-Capuska et al., 2014). Hence, the results of the present study suggest a constraint may be limiting the foraging range of gannets from Pope's Eye.

**Fig. 2.**
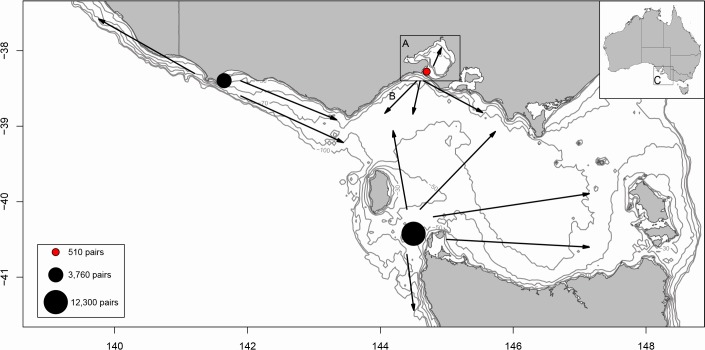
**Location of Pope's Eye gannet colony.** The colony is indicated by the red dot in south-eastern Australia (dark rectangle on inset map A). Environmental data was extracted from (A) Port Phillip Bay and (B) Bass Strait. In addition, environmental data were obtained in the region of the seasonally active (C) Bonney Upwelling (shown in inset map C). Bathymetry and edge of the continental shelf are indicated by light grey lines. Other gannet colonies in the region are indicated by a black dot (proportional to their size, as detailed in the legend). Colony size for Pope's Eye includes all birds nesting in Port Phillip Bay due to their close proximity. As birds typically remain on the continental shelf to forage, arrows indicate potential foraging areas and direction for each gannet colony, based on colony size and location (Wakefield et al., 2013).

Intra-specific competition may also influence the distance and duration a marine predator forages (Lewis et al., 2001). Indeed, density-dependent competition has been shown in Cape and northern gannets to create mutually exclusive foraging areas between adjacent colonies (Grémillet et al., 2004; Wakefield et al., 2013). Pope's Eye colony is comprised of up to 180 breeding pairs, with an additional 330 nests scattered throughout Port Phillip Bay (Pyk et al., 2013). Two other major Australasian gannet colonies occur in relatively close proximity: Lawrence Rocks (3100 pairs) and its sub-colony Point Danger (660 pairs) in western Bass Strait; and Black Pyramid (12,300 pairs) in southern Bass Strait (Bunce et al., 2002). As gannets typically remain on the continental shelf to forage (location of preferred prey; Fletcher and Tregonning, 1992), these much larger colonies are likely to have established mutually exclusive foraging zones stretching into Bass Strait, potentially restricting the foraging range of individuals from the Pope's Eye colony (Fig. 2). Consequently, in years of reduced local prey availability, individuals from Pope's Eye may not be able to extend their foraging range due to intra-specific competition (Wakefield et al., 2013). Such competition could potentially lead to a reduced foraging range if individuals from other larger colonies extend their movements in search of prey in times of reduce availability.

As gannets forage in three-dimensions, the effort involved in resource acquisition may not be accurately reflected from horizontal movements alone (Shepard et al., 2008). In the present study, the proportion of foraging trips spent resting on the sea surface were similar between years and consistent with previous reports from the same colony (Green et al., 2010). However, mean VeDBA and total VeDBA (an index of energy expenditure; Gleiss et al., 2011; Qasem et al., 2012) was significantly greater in years of lower apparent food availability suggesting the birds were working harder searching for prey (Shepard et al., 2008). Indeed, the proportion of time spent foraging in a trip across both sexes and stages was found to be higher in 2012 compared to 2011, increasing their foraging when prey availability was apparently low. Foraging time included both plunge diving (used to calculate dive rate) and surface foraging, a foraging event consuming a high percent of the foraging time (Garthe et al., 2000; Warwick-Evans et al., 2015). While the energetic cost of plunge diving is considered relatively low, the effort involved in taking-off from the water surface may be substantial (Green et al., 2010). Indeed, the combination of increased foraging and flapping flight for take-off may be causal to the increase in mean VeDBA across years. As the proportion of time spent foraging did not significantly increase from 2012 to 2013, birds may have reached their limit for energy expended.

To cover a range of temporal restrictions on the foraging adult, two stages of breeding were investigated; incubation and chick rearing. During incubation adults take turns guarding the nest with only self-maintenance and the fasting ability of the partner driving foraging decisions. During chick rearing, the duration of foraging trips reduces due to the chicks' fasting ability, and chick-maintenance becomes important for foraging decisions. Interestingly, regardless of breeding stage, both males and females increased the proportion of the trip foraging and both proxies of energy expenditure (total and mean VeDBA) from 2011 to 2012, presumably a year of poor prey availability. As a long lived species, increased effort in poor conditions will only occur until a certain threshold, which appears to have been encountered in 2013 when only 10 chicks were raised to fledging age from the 116 nests which attempted to breed.

The results of the present study, therefore, suggest that Australasian gannets from the Pope's Eye colony did not extend their foraging range in response to reduced prey availability but rather increased foraging effort within their normal range, potentially restricted by conspecifics at nearby colonies. However, as evidenced by the reduced fledging success, an increased foraging effort was not sufficient to compensate for the supposed reduction in prey availability. It should be noted that these findings stem from analysis of the first trip of each individual and a low sample size in incubation of 2011. Hence, these results should be interpreted with caution. However, these findings highlight the potential for intra-specific density-dependent competition within meta-populations to potentially limit the ability of component sub-populations to adjust behaviour in response to environmental variability. With predictions of increased sea surface temperatures and other physical changes to the ocean (IPCC, 2013), knowledge of how seabirds can adapt (Sydeman et al., 2012) and the factors limiting those adaptations are essential for effective management of seabird populations in the future.

## MATERIALS AND METHODS

### Study site and animal handling

The study was conducted between October and February, over three consecutive breeding seasons (2011–2013) at the Pope's Eye gannet colony (38°16′42″S 144°41′48″E; Fig. 2), in south-eastern Australia. Sampling was separated into two stages, incubation and chick rearing. During incubation, gannets take turns to guard the egg while their partner forages. Whereas during chick rearing, adults continue to alternate remaining at the nest but obtain enough food for the chick and self-provisioning. Hence, the two stages cover the different time restrictions faced by a foraging adult due to the fasting capabilities of its chick and breeding partner.

Study nests were selected at random throughout the colony in each of these stages and, where possible, both partners were sampled to ensure an equal sex ratio. All nests in the colony were monitored fortnightly for the duration of the breeding season. Breeding success was determined by the percentage of laid eggs which became fledged chicks. Chicks sighted for 90 consecutive days since hatching and displaying juvenile plumage were presumed to have fledged (Pyk et al., 2007).

Adults were captured on the nest and the egg/chick covered for protection from aggressive conspecifics on neighbouring nests. All animal handling followed protocols approved by Deakin University Animal Welfare Committee [A86/2010] and Department of Sustainability and Environment Victoria Wildlife Research [Permit 0005745]. In order to determine the at-sea movements of breeding birds, individuals were equipped with a GPS data logger (IgotU GT-600, Mobile Action Technologies Inc., Taiwan; 26.5 g) recording location (±10 m) every 2 min. In addition, to obtain information about at-sea activity patterns and foraging effort (Shepard et al., 2010), all individuals were also instrumented with a tri-axial accelerometer data logger (X8 500mAh, Gulf Coast Data Concepts LLC, USA; 14.1 g) sampling at 25 Hz. The devices were encapsulated in heat shrink (whole package 52.6 g) (<3% body mass; Phillips et al., 2003) and attached with water-proof tape (Tesa, Beiersdorf AG, Germany) to the central tail feathers following the methods of Wilson et al. (1997). Device positioning ensured it was covered by the wings during a plunge dive in an attempt to reduce drag (Hamer et al., 2000). Individuals were then weighed in a cloth bag using a suspension scale (±25 g, Salter Australia Pty Ltd, Australia) and morphometric measurements (i.e. bill length and depth, total head, wing and tarsus length) were taken before individuals were returned to the nest with the whole procedure lasting less than 10 min. Individuals were recaptured after 1–12 days, weighed as previously described and the devices removed. Due to logistical constraints, devices were recovered from most individuals beyond the battery life. As such, mass gain by the adult and chick on recovery could not be matched up to the GPS and accelerometry data to infer foraging success. A single blood sample (0.1 ml) was then collected by venepuncture of a tarsus vein for genetic sexing (DNASolutions, Australia) before the individual was released. Individuals were sampled only once in each breeding season.

### Environmental variables and data analysis

To determine if environmental conditions which may influence prey availability varied during the study, weekly sea surface temperatures (SST, °C) and sea-surface chlorophyll-*a* concentrations (chl-*a*, mg m^−3^) were extracted from areas known to be frequented by foraging gannets (T. M. Pyk, PhD thesis, Deakin University, 2012; this study), i.e. Port Phillip Bay (38°23′S–37°47′S; 144°18′E–145°11′E) and Bass Strait (40°12′S - 38°30′; 144°5′E - 146°48′E; Fig. 2). The influence of environmental conditions in the months prior to breeding have previously been found to influence breeding success in seabirds (Thompson and Ollason, 2001; Barbraud and Weimerskirch, 2003). Therefore, conditions prior to breeding commencing (June-August) as well as during the breeding months (October-January) were considered. Additionally, SST and chl-*a* for the nearby Bonney Upwelling (39°S–38°S; 136°E−142°E) (strongest between January and March; Butler et al., 2002; Hobday and Hartog, 2014), an oceanographic feature of ecological importance to Bass Strait (Butler et al., 2002), were obtained. The Bonney Upwelling has been shown to influence the breeding success of fur seals in the subsequent summer (i.e. cascade effect on nutrients; Gibbens and Arnould, 2009) and, thus, may influence prey availability for the Australasian gannet. Therefore, environmental variables from the Bonney Upwelling region in the January-March period prior to the breeding season were investigated. Weekly means for environmental variables were obtained from the AVHRR sensors (SST; resolution 4 km; courtesy of CSIRO Oceans and Atmosphere Remote Sensing) and MODIS satellites (chl-*a*; 4 km; courtesy of NASA, http://oceandata.sci.gsfc.nasa.gov).

GPS locations were processed through a speed filter (McConnell et al., 1992) and summary statistics calculated with the *adehabitatHR* package (Calenge, 2006) in the R statistical environment (R Core Team, 2015). While the number of foraging trips per individual ranged from 1–16 trips, most individuals in the first year of the study only conducted one trip before the batteries failed. Hence, stages were compared between years using only the first foraging trip for each individual. Foraging trip metrics were calculated (i.e. maximum distance from colony, total distance and duration, and average speed) for the foraging trip.

Data obtained from the tri-axial accelerometer were used to visually assess behaviour in IGOR Pro (Version 6.34, WaveMetrics, USA) based on previous studies of plunge diving species (Ropert-Coudert et al., 2004, 2009b; Weimerskirch et al., 2005b). Four key behaviours were identified: resting at the sea surface, flapping flight, gliding flight, and foraging (including plunge diving and surface foraging; Warwick-Evans et al., 2015). The *Ethographer* package was used to perform a *k*-means algorithm clustering analysis (see Sakamoto et al., 2009) and identify behaviour using an unsupervised continuous wavelet transformation (1 s window). Each cluster was assigned a behaviour based on the visual identification. From this, the proportion of time spent performing each behaviour, within a foraging trip, was calculated. Additionally, the total number of dives were used to calculate dive rate (dives h^−1^) averaged over the foraging trip. Overall dynamic body acceleration (ODBA) is typically used as an indication of energetic expenditure obtained from the accelerometry data (Wilson et al., 2006; Gomez Laich et al., 2011; Elliott et al., 2013). However, as the accelerometer could not be placed in the centre of gravity of the bird due to likely removal (Vandenabeele et al., 2014), vectorial dynamic body acceleration (VeDBA) was determined to be more appropriate than ODBA (Gleiss et al., 2011). VeDBA was calculated following the methods outlined by Qasem et al. (2012), the sum of all values (total VeDBA) and the mean VeDBA were then calculated. Total VeDBA was considered as it incorporates the trip duration into the values, whereas mean VeDBA allows comparison of the rate of energy expended across years.

As behavioural data were proportional, an arcsine transformation was performed. The assumptions of independent and normally distributed data were tested with a Chi-Square test and Shapiro–Wilk's test, respectively. Where these assumptions were not met, a log_10_ transformation was performed. Data was analysed using one-way ANOVAs followed by Tukey's post hoc tests to assess inter-annual variation. Data are reported as mean±s.e.m.

## References

[BIO013250C1] Abrams P. A. (1991). Life history and the relationship between food availability and foraging effort. *Ecology* 72, 1242-1252. 10.2307/1941098

[BIO013250C2] Adams E. S. (2001). Approaches to the study of territory size and shape. *Annu. Rev. Ecol. Syst.* 32, 277-303. 10.1146/annurev.ecolsys.32.081501.114034

[BIO013250C3] Barbraud C. and Weimerskirch H. (2003). Climate and density shape population dynamics of a marine top predator. *Proc. R. Soc. B Biol. Sci.* 270, 2111-2116. 10.1098/rspb.2003.2488 PMC1691492 14561273

[BIO013250C4] Berlincourt M. and Arnould J. P. Y. (2015a). Influence of environmental conditions on foraging behaviour and its consequences on reproductive performance in little penguins. *Mar. Biol.* 162, 1485-1501. 10.1007/s00227-015-2685-x

[BIO013250C5] Berlincourt M. and Arnould J. P. Y. (2015b). Breeding short-tailed shearwaters buffer local environmental variability in south-eastern Australia by foraging in Antarctic waters. *Mov. Ecol.* 3, 16 10.1186/s40462-015-0044-7 26236479 PMC4522076

[BIO013250C6] Bunce A. (2001). Prey consumption of Australasian gannets (*Morus serrator*) breeding in Port Phillip Bay, southeast Australia, and potential overlap with commercial fisheries. *ICES J. Mar. Sci.* 58, 904-915. 10.1006/jmsc.2001.1083

[BIO013250C7] Bunce A. and Norman F. I. (2000). Changes in the diet of the Australasian gannet (Morus serrator) in response to the 1998 mortality of pilchards (*Sardinops sagax*). *Mar. Freshwater Res.* 51, 349-353. 10.1071/MF99133

[BIO013250C8] Bunce A., Norman F. I., Brothers N. and Gales R. (2002). Long-term trends in the Australasian gannet (*Morus serrator*) population in Australia: the effect of climate change and commercial fisheries. *Mar. Biol.* 141, 263-269. 10.1007/s00227-002-0838-1

[BIO013250C9] Burke C. M. and Montevecchi W. A. (2009). The foraging decisions of a central place foraging seabird in response to fluctuations in local prey conditions. *J. Zool.* 278, 354-361. 10.1111/j.1469-7998.2009.00584.x

[BIO013250C10] Butler A., Althaus F., Furlani D. and Ridgway K. (2002). *Assessment of the Conservation Values of the Bonney Upwelling Area: A Component of the Commonwealth Marine Conservation Assessment Program 2002–2004*, (ed. D.o.t. Environment). Information Solution Works, Hobart, Tasmania: CSIRO Marine Research.

[BIO013250C11] Cairns D. K. (1987). Seabirds as indicators of marine food supplies. *Biol. Oceanogr.* 5, 261-271.

[BIO013250C12] Calenge C. (2006). The package “adehabitat” for the R software: a tool for the analysis of space and habitat use by animals. *Ecol. Model.* 197, 516-519. 10.1016/j.ecolmodel.2006.03.017

[BIO013250C13] Clutton-Brock T. H. (1991). *The Evolution of Parental Care*. Princeton, New Jersey: Princeton University Press.

[BIO013250C14] Cullen J. M., Chambers L. E., Coutin P. C. and Dann P. (2009). Predicting onset and success of breeding in little penguins Eudyptula minor from ocean temperatures. *Mar. Ecol. Prog. Ser.* 378, 269-278. 10.3354/meps07881

[BIO013250C15] Edmunds M. J. (2003). *Victorian Subtidal Reef Monitoring Program: The Reef Biota At Port Phillip Heads Marine National Park*, pp. 269-278. Melbourne, Victoria: Parks Victoria.

[BIO013250C16] Elliott K. H., Le Vaillant M., Kato A., Speakman J. R. and Ropert-Coudert Y. (2013). Accelerometry predicts daily energy expenditure in a bird with high activity levels. *Biol. Lett.* 9, 1-4. 10.1098/rsbl.2012.0919 PMC3565507 23256182

[BIO013250C17] Erikstad K. E., Fauchald P., Tveraa T. and Steen H. (1998). On the cost of reproduction in long-lived birds: the influence of environmental variability. *Ecology* 79, 1781-1788. 10.1890/0012-9658(1998)079[1781:OTCORI]2.0.CO;2

[BIO013250C18] Fletcher W. J. and Tregonning R. J. (1992). Distribution and timing of spawning by the Australian pilchard (*Sardinops sagax neopilchardus*) off Albany, Western Australia. *Mar. Freshwater Res.* 43, 1437-1449. 10.1071/MF9921437

[BIO013250C19] Frederiksen M., Harris M. P., Daunt F., Rothery P. and Wanless S. (2004). Scale-dependent climate signals drive breeding phenology of three seabird species. *Global Change Biol.* 10, 1214-1221. 10.1111/j.1529-8817.2003.00794.x

[BIO013250C20] Garthe S., Benvenuti S. and Montevecchi W. A. (2000). Pursuit plunging by northern gannets (*Sula bassana*) “feeding on capelin (*Mallotus villosus*)”. *Proc. R. Soc. B Biol. Sci.* 267, 1717-1722. 10.1098/rspb.2000.1200 PMC1690745 12233767

[BIO013250C21] Gibbens J. and Arnould J. P. Y. (2009). Interannual variation in pup production and the timing of breeding in benthic foraging Australian fur seals. *Mar. Mamm. Sci.* 25, 573-587. 10.1111/j.1748-7692.2008.00270.x

[BIO013250C22] Gibbs C. F. (1992). Oceanography of Bass Strait: implications for the food supply of Little Penguins *Eudyptula minor*. *Emu* 91, 395-401. 10.1071/MU9910395

[BIO013250C23] Gleiss A. C., Wilson R. P. and Shepard E. L. C. (2011). Making overall dynamic body acceleration work: on the theory of acceleration as a proxy for energy expenditure. *Methods Ecol. Evol.* 2, 23-33. 10.1111/j.2041-210X.2010.00057.x

[BIO013250C24] Gomez Laich A., Wilson R. P., Gleiss A. C., Shepard E. L. C. and Quintana F. (2011). Use of overall dynamic body acceleration for estimating energy expenditure in cormorants: does locomotion in different media affect relationships? *J. Exp. Mar. Biol. Ecol.* 399, 151-155. 10.1016/j.jembe.2011.01.008

[BIO013250C25] Grecian W. J., Witt M. J., Attrill M. J., Bearhop S., Godley B. J., Grémillet D., Hamer K. C. and Votier S. C. (2012). A novel projection technique to identify important at-sea areas for seabird conservation: an example using Northern gannets breeding in the North East Atlantic. *Biol. Conserv.* 156, 43-52. 10.1016/j.biocon.2011.12.010

[BIO013250C26] Green J. A., White C. R., Bunce A., Frappell P. B. and Butler P. J. (2010). Energetic consequences of plunge diving in gannets. *Endang. Species Res.* 10, 269-279. 10.3354/esr00223

[BIO013250C27] Grémillet D., Dell'Omo G., Ryan P. G., Peters G., Ropert-Coudert Y. and Weeks S. J. (2004). Offshore diplomacy or how seabirds mitigate intra-specific competition: a case study based on GPS tracking of Cape gannets from neighbouring colonies. *Mar. Ecol. Prog. Ser.* 268, 265-279. 10.3354/meps268265

[BIO013250C28] Hamer K. C., Monaghan P., Uttley J. D., Walton P. and Burns M. D. (1993). The influence of food supply on the breeding ecology of Kittiwakes Rissa tridactyla in Shetland. *Ibis* 135, 255-263. 10.1111/j.1474-919X.1993.tb02842.x

[BIO013250C29] Hamer K. C., Phillips R. A., Wanless S., Harris M. P. and Wood A. G. (2000). Foraging ranges, diets and feeding locations of gannets Morus bassanus in the North Sea: evidence from satellite telemetry. *Mar. Ecol. Prog. Ser.* 200, 257-264. 10.3354/meps200257

[BIO013250C30] Hamer K. C., Humphreys E. M., Garthe S., Hennicke J., Peters G., Grémillet D., Phillips R. A., Harris M. P. and Wanless S. (2007). Annual variation in diets, feeding locations and foraging behaviour of gannets in the North Sea: flexibility, consistency and constraint. *Mar. Ecol. Prog. Ser.* 338, 295-305. 10.3354/meps338295

[BIO013250C31] Hennicke J. C. and Weimerskirch H. (2014). Coping with variable and oligotrophic tropical waters: foraging behaviour and flexibility of the Abbott's booby *Papasula abbotti*. *Mar. Ecol. Prog. Ser.* 499, 259-273. 10.3354/meps10664

[BIO013250C32] Hobday A. J. and Hartog J. R. (2014). Using dynamic ocean features in ocean management. *Oceanography* 27, 134-145. 10.5670/oceanog.2014.92

[BIO013250C33] Hoskins A. J. and Arnould J. P. Y. (2014). Relationship between long-term environmental fluctuations and diving effort of female Australian fur seals. *Mar. Ecol. Prog. Ser.* 511, 285-295. 10.3354/meps10935

[BIO013250C34] Inchausti P., Guinet C., Koudil M., Durbec J.-P., Barbraud C., Weimerskirch H., Cherel Y. and Jouventin P. (2003). Inter-annual variability in the breeding performance of seabirds in relation to oceanographic anomalies that affect the Crozet and the Kerguelen sectors of the Southern Ocean. *J. Avian Biol.* 34, 170-176. 10.1034/j.1600-048X.2003.03031.x

[BIO013250C35] IPCC (2013). Observations: ocean. In *Climate Change 2013: The Physical Science Basis. Contribution of Working Group I to the Fifth Assessment Report of the Intergovernmental Panel on Climate Change*. (ed. H. Freeland, S. Garzoli and Y. Nojiri) Cambridge, NY, USA: Cambridge University Press.

[BIO013250C36] Knox T. C., Stuart-Williams H., Warneke R. M., Hoskins A. J. and Arnould J. P. Y. (2014). Analysis of growth and stable isotopes in teeth of male Australian fur seals reveals interannual variability in prey resources. *Mar. Mamm. Sci.* 30, 763-781. 10.1111/mms.12078

[BIO013250C37] Lewis S., Sherratt T. N., Hamer K. C. and Wanless S. (2001). Evidence of intra-specific competition for food in a pelagic seabird. *Nature* 412, 816-819. 10.1038/35090566 11518965

[BIO013250C38] Ludynia K., Kemper J. and Roux J.-P. (2012). The Namibian Islands’ Marine Protected Area: using seabird tracking data to define boundaries and assess their adequacy. *Biol. Conserv.* 156, 136-145. 10.1016/j.biocon.2011.11.014

[BIO013250C39] Machovsky-Capuska G. E., Hauber M. E., Libby E., Amiot C. and Raubenheimer D. (2014). The contribution of private and public information in foraging by Australasian gannets. *Anim. Cogn.* 17, 849-858. 10.1007/s10071-013-0716-x 24337907

[BIO013250C40] McConnell B. J., Chambers C. and Fedak M. A. (1992). Foraging ecology of southern elephant seals in relation to the bathymetry and productivity of the Southern Ocean. *Antarct. Sci.* 4, 393-398. 10.1017/S0954102092000580

[BIO013250C41] Mickelson M. J., Dann P. and Cullen J. M. (1992). Sea temperature in Bass Strait and breeding success of the little penguin *Eudyptula minor* at Phillip Island, south-eastern Australia. *Emu* 91, 355-368. 10.1071/MU9910355

[BIO013250C42] Monaghan P., Walton P., Wanless S., Uttley J. D. and Bljrns M. D. (1994). Effects of prey abundance on the foraging behaviour, diving efficiency and time allocation of breeding Guillemots Uria aalge. *Ibis* 136, 214-222. 10.1111/j.1474-919X.1994.tb01087.x

[BIO013250C43] Montevecchi W. A. (1993). Birds as indicators of change in marine prey stocks. In *Birds as Monitors of Environmental Change* (ed. Furness R. W. and Greenwood J. J. D.), pp. 217-266. Houten: Springer Netherlands.

[BIO013250C44] Monticelli D., Ramos J. A. and Quartly G. D. (2007). Effects of annual changes in primary productivity and ocean indices on breeding performance of tropical roseate terns in the western Indian Ocean. *Mar. Ecol. Prog. Ser.* 351, 273-286. 10.3354/meps07119

[BIO013250C45] Nelson J. B. (1978). *The Sulidae: Gannets and Boobies*. London: Oxford University Press.

[BIO013250C46] Nieblas A.-E., Sloyan B. M., Hobday A. J., Coleman R. and Richardson A. J. (2009). Variability of biological production in low wind-forced regional upwelling systems: a case study off southeastern Australia. *Limnol. Oceanogr.* 54, 1548-1558. 10.4319/lo.2009.54.5.1548

[BIO013250C47] Norman F. I., Minton C. D. T., Bunce A. and Govanstone A. P. (1998). Recent changes in the status of Australasian Gannets *Morus serrator* in Victoria. *Emu* 98, 147-150. 10.1071/MU98018

[BIO013250C48] Orians G. H. and Pearson N. E. (1979). *On the Theory of Central Place Foraging*. Ohio: Ohio State University Press.

[BIO013250C49] Phillips R. A., Xavier J. C. and Croxall J. P. (2003). Effects of satellite transmitters on albatrosses and petrels. *Auk* 120, 1082-1090. 10.1642/0004-8038(2003)120[1082:EOSTOA]2.0.CO;2

[BIO013250C50] Pichegru L., Ryan P. G., van der Lingen C. D., Coetzee J., Ropert-Coudert Y. and Grémillet D. (2007). Foraging behaviour and energetics of Cape gannets *Morus capensis* feeding on live prey and fishery discards in the Benguela upwelling system. *Mar. Ecol. Prog. Ser.* 350, 127-136. 10.3354/meps07128

[BIO013250C51] Pichegru L., Ryan P. G., Crawford R. J. M., van der Lingen C. D. and Grémillet D. (2010). Behavioural inertia places a top marine predator at risk from environmental change in the Benguela upwelling system. *Mar. Biol.* 157, 537-544. 10.1007/s00227-009-1339-2

[BIO013250C52] Poloczanska E. S., Babcock R. C., Butler A., Hobday A. J., Hoegh-Guldberg O., Kunz T. J., Matear R., Milton D. A., Okey T. A. and Richardson A. J. (2007). Climate change and Australian marine life. *Oceanogr. Mar. Biol.* 45, 407-478.

[BIO013250C54] Pyk T. M., Bunce A. and Norman F. I. (2007). The influence of age on reproductive success and diet in Australasian Gannets (*Morus serrator*) breeding at Pope's Eye, Port Phillip Bay, Victoria. *Aust. J. Zool.* 55, 267-274. 10.1071/ZO06088

[BIO013250C55] Pyk T. M., Weston M. A., Bunce A. and Norman F. I. (2013). Establishment and development of a seabird colony: long-term trends in phenology, breeding success, recruitment, breeding density and demography. *J. Ornithol.* 154, 299-310. 10.1007/s10336-012-0894-3

[BIO013250C56] Pyke G. H., Pulliam H. R. and Charnov E. L. (1977). Optimal foraging: a selective review of theory and tests. *Q. Rev. Biol.* 52, 137-154. 10.1086/409852

[BIO013250C57] Qasem L., Cardew A., Wilson A., Griffiths I., Halsey L. G., Shepard E. L. C., Gleiss A. C. and Wilson R. (2012). Tri-axial dynamic acceleration as a proxy for animal energy expenditure; should we be summing values or calculating the vector? *PLoS ONE* 7, e31187 10.1371/journal.pone.0031187 22363576 PMC3281952

[BIO013250C58] R Core Team (2015). *R: A Language and Environment for Statistical Computing*. Vienna, Austria: R Foundation for Statistical Computing.

[BIO013250C59] Ricklefs R. E., Day C. H., Huntington C. E. and Williams J. B. (1985). Variability in feeding rate and meal size of Leach's storm-petrel at Kent Island, New Brunswick. *J. Anim. Ecol.* 54, 883-898. 10.2307/4385

[BIO013250C60] Ridgway K. and Hill K. (2009). The East Australian current. In *A Marine Climate Change Impacts and Adaptation Report Card for Australia 2009* (ed. Poloczanska E. S., Hobday A. J. and Richardson A. J.), pp. 1-16. Brisbane, Queensland: NCCARF Publication.

[BIO013250C61] Ronconi R. A. and Burger A. E. (2008). Limited foraging flexibility: increased foraging effort by a marine predator does not buffer against scarce prey. *Mar. Ecol. Prog. Ser.* 366, 245-258. 10.3354/meps07529

[BIO013250C62] Ropert-Coudert Y., Grémillet D., Ryan P., Kato A., Naito Y. and Le Maho Y. (2004). Between air and water: the plunge dive of the Cape gannet *Morus capensis*. *Ibis* 146, 281-290. 10.1111/j.1474-919x.2003.00250.x

[BIO013250C63] Ropert-Coudert Y., Kato A. and Chiaradia A. (2009a). Impact of small-scale environmental perturbations on local marine food resources: a case study of a predator, the little penguin. *Proc. R. Soc. B Biol. Sci.* 276, 4105-4109. 10.1098/rspb.2009.1399 PMC2821355 19729454

[BIO013250C64] Ropert-Coudert Y., Daunt F., Kato A., Ryan P. G., Lewis S., Kobayashi K., Mori Y., Grémillet D. and Wanless S. (2009b). Underwater wingbeats extend depth and duration of plunge dives in northern gannets *Morus bassanus*. *J. Avian Biol.* 40, 380-387. 10.1111/j.1600-048X.2008.04592.x

[BIO013250C65] Sakamoto K. Q., Sato K., Ishizuka M., Watanuki Y., Takahashi A., Daunt F. and Wanless S. (2009). Can ethograms be automatically generated using body acceleration data from free-ranging birds? *PLoS ONE* 4, e5379 10.1371/journal.pone.0005379 19404389 PMC2671159

[BIO013250C66] Sandvik H., Erikstad K. E. and Sæther B. E. (2012). Climate affects seabird population dynamics both via reproduction and adult survival. *Mar. Ecol. Prog. Ser.* 454, 273-284. 10.3354/meps09558

[BIO013250C67] Shepard E. L. C., Wilson R. P., Halsey L. G., Quintana F., Gomez Laich A., Gleiss A. C., Liebsch N., Myers A. E. and Norman B. (2008). Derivation of body motion via appropriate smoothing of acceleration data. *Aquat. Biol.* 4, 235-241. 10.3354/ab00104

[BIO013250C68] Shepard E. L. C., Wilson R. P., Quintana F., Gomez Laich A., Liebsch N., Albareda D. A., Halsey L. G., Gleiss A., Morgan D. T., Myers A. E. et al. (2010). Identification of animal movement patterns using tri-axial accelerometry. *Endang. Species Res.* 10, 47-60. 10.3354/esr00084

[BIO013250C69] Stenseth N. C., Mysterud A., Ottersen G., Hurrell J. W., Chan K.-S. and Lima M. (2002). Ecological effects of climate fluctuations. *Science* 297, 1292-1296. 10.1126/science.1071281 12193777

[BIO013250C70] Sydeman W. J., Thompson S. A. and Kitaysky A. (2012). Seabirds and climate change: roadmap for the future. *Mar. Ecol. Prog. Ser.* 454, 107-117. 10.3354/meps09806

[BIO013250C71] Thompson P. M. and Ollason J. C. (2001). Lagged effects of ocean climate change on fulmar population dynamics. *Nature* 413, 417-420. 10.1038/35096558 11574887

[BIO013250C72] Vandenabeele S. P., Grundy E., Friswell M. I., Grogan A., Votier S. C. and Wilson R. P. (2014). Excess baggage for birds: inappropriate placement of tags on gannets changes flight patterns. *PLoS ONE* 9, e92657 10.1371/journal.pone.0092657 24671007 PMC3966804

[BIO013250C73] Votier S. C., Bicknell A., Cox S. L., Scales K. L. and Patrick S. C. (2013). A bird's eye view of discard reforms: bird-borne cameras reveal seabird/fishery interactions. *PLoS ONE* 8, e57376 10.1371/journal.pone.0057376 23483906 PMC3590202

[BIO013250C74] Wakefield E. D., Bodey T. W., Bearhop S., Blackburn J., Colhoun K., Davies R., Dwyer R. G., Green J. A., Grémillet D., Jackson A. L. et al. (2013). Space partitioning without territoriality in gannets. *Science* 341, 68-70. 10.1126/science.1236077 23744776

[BIO013250C75] Wanless S., Harris M. P., Redman P. and Speakman J. R. (2005). Low energy values of fish as a probable cause of a major seabird breeding failure in the North Sea. *Mar. Ecol. Prog. Ser.* 294, 1-8. 10.3354/meps294001

[BIO013250C76] Warwick-Evans V., Atkinson P. W., Gauvain R. D., Robinson L. A., Arnould J. P. Y. and Green J. A. (2015). Time-in-area represents foraging activity in a wide-ranging pelagic forager. *Mar. Ecol. Prog. Ser.* 527, 233-246. 10.3354/meps11262

[BIO013250C77] Watanuki Y. and Ito M. (2012). Climatic effects on breeding seabirds of the northern Japan Sea. *Mar. Ecol. Prog. Ser.* 454, 183-196. 10.3354/meps09627

[BIO013250C78] Weimerskirch H. (2007). Are seabirds foraging for unpredictable resources? *Deep Sea Res. Part II Top. Stud. Oceanogr.* 54, 211-223. 10.1016/j.dsr2.2006.11.013

[BIO013250C79] Weimerskirch H., Mougey T. and Hindermeyer X. (1997). Foraging and provisioning strategies of black-browed albatrosses in relation to the requirements of the chick: natural variation and experimental study. *Behav. Ecol.* 8, 635-643. 10.1093/beheco/8.6.635

[BIO013250C80] Weimerskirch H., Le Corre M., Jaquemet S. and Marsac F. (2005a). Foraging strategy of a tropical seabird, the red-footed booby, in a dynamic marine environment. *Mar. Ecol. Prog. Ser.* 288, 251-261. 10.3354/meps288251

[BIO013250C81] Weimerskirch H., Le Corre M., Ropert-Coudert Y., Kato A. and Marsac F. (2005b). The three-dimensional flight of red-footed boobies: adaptations to foraging in a tropical environment? *Proc. R. Soc. B Biol. Sci.* 272, 53-61. 10.1098/rspb.2004.2918 PMC1634943 15875570

[BIO013250C82] Wilson R. P., Pütz K., Peters G., Culik B., Scolaro J. A., Charrassin J. B. and Ropert-Coudert Y. (1997). Long-term attachment of transmitting and recording devices to penguins and other seabirds. *Wildl. Soc. Bull.* 25, 101-105.

[BIO013250C83] Wilson R. P., White C. R., Quintana F., Halsey L. G., Liebsch N., Martin G. R. and Butler P. J. (2006). Moving towards acceleration for estimates of activity-specific metabolic rate in free-living animals: the case of the cormorant. *J. Anim. Ecol.* 75, 1081-1090. 10.1111/j.1365-2656.2006.01127.x 16922843

